# The Influence of Affective State on Subjective-Report Measurements: Evidence From Experimental Manipulations of Mood

**DOI:** 10.3389/fpsyg.2021.601083

**Published:** 2021-02-18

**Authors:** Kine Askim, Stein Knardahl

**Affiliations:** National Institute of Occupational Health, Oslo, Norway

**Keywords:** subjective reports, self-report, method bias, affective state, mood induction, questionnaires

## Abstract

A substantial portion of the knowledge base of psychology is based on subjective reports with a risk of information bias. The objective of the present study was to elucidate one contextual source of variance and potential bias in subjective reports: the influence of affective state at the time of responding to questionnaires. Employees (*N* = 67, abstaining from stimulants and activities that may influence emotional and physiological state) were subjected to mood-induction procedures in the laboratory. Neutral, positive, and negative moods were induced by combinations of pictures from the international affective picture set (IAPS) and music. The subjects responded to questions on visual analog scales (VAS) in order to optimize sensitivity and attenuate short-term memory effects. Most subjects exhibited significant affective-state inductions with no change in arousal. The analyses took affective response to the manipulation into account. Only four of 20 questions were somewhat influenced by induced affective state: job overload, social support from co-workers, satisfaction with getting to develop personally, and an item measuring agreeableness. In general, responding to questions of work that were phrased for valence was little or insignificantly influenced by induced affective state.

## Introduction

A substantial portion of the knowledge base of industrial, organizational, and health psychology is based on reports by individual subjects of their perception and appraisal of the phenomena under study, i.e., subjective reports (Bodner, 2006). Furthermore, surveys are perhaps the most frequently applied data collection technique used by organizations (Rivers et al., 2009). However, subjective reports are commonly considered with skepticism. A major concern with methods based on subjective reports is the risk of method bias, i.e., method factors that influence the subject’s responding, thereby introducing method variance and/or bias of estimates of the construct (trait) that is measured [see Podsakoff et al. (2012) for an excellent review and McCrae (2018) for method biases in personality assessment]. In particular, the assumption that associations based on same-source, self-reported data are inherently invalid due to common method variance (CMV) or common method bias (CMB) has received much attention (e.g., Conway and Lance, 2010; Fuller et al., 2016; Wingate et al., 2018).

Answering questions involve several cognitive processes: interpreting the meaning and intent of the question, searching memory for information, forming a judgment, and translating the judgment into a response given the response categories presented. There are potential sources of subjective-report method bias despite adequate psychometric validation of instruments: (I) Personality characteristics of the individual may influence mechanisms for perception and appraisal and hence possibly influence the reporting of almost all environmental exposures, situations, states, somatic sensations, and symptoms. There is a large body of studies showing that neuroticism and negative-affectivity trait predispose for reporting mental and somatic symptoms (e.g., Cuijpers et al., 2010; Vassend et al., 2018). Neuroticism also seems to influence the reporting of perceived social support in undergraduate students (Swickert and Owens, 2010). Moreover, the social-desirability (self-deception) trait may produce bias by systematic over- or underreporting according to social norms (e.g., Nederhof, 1985). (II) Response styles are “tendencies to respond systematically to questionnaire items on some basis other than what the items were specifically designed to measure” (Baumgartner and Steenkamp, 2001). Well-documented examples are acquiescence responding (the tendency to agree with items regardless of content; Knowles and Condon, 1999), extreme response style (the tendency to endorse the most extreme response categories), and midpoint responding (the tendency to use the middle category regardless of content). Some of these strategies may be due to the use of heuristics to minimize cognitive effort. (III) Instrument-design effects influence subjective reports due to the wording of questions and response alternatives or the influence of one set of questions on those following (consistency or carryover effects; for a condensed guide to questionnaire design, see Krosnick and Presser, 2010). Finally, (IV) context factors at the time of responding to the questionnaire may influence affective state, situation models, and cognitive representation. Fleeson (2001) found that measures of personality traits varied with time-of-day and the number of others. Previous experimental studies suggest that mood may influence the reporting of health and social support (Croyle and Uretsky, 1987; Cohen et al., 1988) as well as measurements of personality (e.g., Reich et al., 1986; Lewis et al., 1995).

The objective of the present study was to elucidate affect as a contextual source of potential method bias inherent in subjective reports, specifically the influence of the subject’s affective state at the time of responding on subjective reports.

Measurements based on subjective reports are used extensively for several reasons: for some factors there are no alternatives (subjective states can only be accessed with subjective reports), questionnaires allow measurements without the influence of an interviewer or observer, they are economical compared to observation methods or objective measurements, and they allow collections of data from large numbers of subjects. Furthermore, for studies of psychological factors one may argue that situations must be perceived by the individual to be of significance. The widely acclaimed transactional theory of coping with challenge (Lazarus and Folkman, 1984) maintains that subjective perception- and appraisal-processes play a pivotal role in determining coping and responding to challenge. Following this reasoning, tapping the subject’s report of his/her appraisal of a situation seems highly relevant for assessing its potential significance. Measuring the appraisal and meaning of a situation by subjective reports purports to measure aspects of the subject’s responses to the situation, i.e., causal sequences in the pathway to outcomes.

The problem of bias relates to the question being asked: If the aim is to determine which factors contribute to motivation, well-being, health, or function in individuals, the individual perception and appraisal are coping mechanisms that play a role in the causal pathway. That is, subjective appraisal is a mediator in causal processes rather than an error. However, transient influences that perturb the recall or representation of one’s appraisal, introduce variance and possibly bias.

If on the other hand the aim is to generate information of the *objective reality*, e.g., job demands or social support, any deviation in subjective reports from those obtained by objective methods is a bias. In this case, validity of the subjective report measurement depends on the correspondence with an objective measurement of the exposure. Scientists have sought to eliminate bias by substituting subjective individual reports with (a) expert assessments of exposures (e.g., job-exposure matrix), (b) group-level data (for each individual use mean of reports from all persons in each unit minus that of the individual), or (c) some objective proxy for the variable under study (e.g., number of units produced as a proxy for job demands). While some studies found similar results with analyses based on individual subjective reports as with analyses based on externally assessed data (e.g., North et al., 1996), group-level data (e.g., Kivimäki et al., 2003; Elovainio et al., 2013), or objective assessments (Hackman and Oldham, 1975; Fried and Ferris, 1987; Spector and Jex, 1991), others found that results based on individual subjective reports of work were not reproduced with objective methods (e.g., Kouvonen et al., 2008).

It seems plausible that *affective state* primarily influences the reporting of factors that imply affective valence. Many aspects of work and social relations imply affective valence. For instance, work tasks may be perceived as interesting, stimulating, etc (positive) or boring, “stressful,” etc (negative). Job satisfaction is a measure of affective valence in addition to cognitive appraisals of input and output (Ilies and Judge, 2004). However, even if questions seem formulated with neutral wording, there is a possibility that affective state may bias responses.

The potential effect of contextual factors influencing affective state at the time of responding on subjective reporting is sparsely discussed in reviews of method bias (e.g., Podsakoff et al., 2012), possibly due to the paucity of relevant studies. Harrison et al. (1996) found that negative perceptions of one factor influenced the reporting of a subsequent factor. They also noted that “A notable minority of subjects was also overtly agitated by the repetition of content in survey questions.” Affective state during retrieval of autobiographical memory may influence the information that is recalled (Buchanan, 2007; Kensinger, 2009). Cohen et al. (1988) reported that *induced mood* influenced recall of self-reported negative life events and on perceived social support (material aid, someone to talk to, others one can do things with, positive comparison with others) in college undergraduates. Therefore, we find it pertinent to address the hypothesis that affective state during responding to a questionnaire may be a contextual factor of significance to method effects.

At any point in time, some participants of a study will be in transient positive moods and others will be in transient negative moods, so if affective states influence responding, the result could be random error and inflated estimates of variance. However, when mood or affective tone generalize to a group or considerable part of an organization, the information gathered from questionnaires may be biased. The contextual factors of an organization or unit may influence the mood of its members (e.g., employees) by perceptions and appraisal of how the organization handles challenges. Contexts like uncertainty, rumors, or threats of downsizing may influence affective states of a large number of employees. Several studies have shown that mood is transferred among individuals in a group (“emotional contagion”; Barsade, 2002; Barsade and Gibson, 2012) and the mood of leaders influence the affective tone of group members (“group affective tone”; Sy et al., 2005; Bono and Ilies, 2006). Moreover, perceptions of politics at organizational levels (others pursuing egocentric goals) and faith in the management may influence depressed mood at work (Byrne et al., 2005). Hence, it is possible that the context of the organization and/or context during the administration of questionnaires can influence the affective state of a large body of study participants. If affective states at the time of assessments influence responding, the result may be systematic errors of measurements.

Affective state can be manipulated in standardized ways with several methods. Music (Västfjäll, 2001-2002), pictures (International Affective Picture System; Horjales-Araujo et al., 2013), film (Croyle and Uretsky, 1987), and combinations of stimuli or tasks (Martin, 1990) can produce significant changes in affective state. Manipulation of affective state may alter pain reports (Wiech and Tracey, 2009; Horjales-Araujo et al., 2013), somatic symptoms (Constantinou et al., 2013), while studies of effects on subjective reports of general health are equivocal (Croyle and Uretsky, 1987; Abele and Hermer, 1993; Barger et al., 2007). Hence, affect may influence several types of judgment (Forgas, 1995).

The overarching aim of the present experimental study was to determine if transient affective states produce a mood-related response bias by influencing how subjects respond to questions pertaining to work, subjective health complaints, and personality traits. Specifically, we manipulated transient affective states with music plus pictures from the International Affective Picture System (IAPS, Lang, 2005). In order to maximize sensitivity and attenuate effects of short-term recall, the subjects rated their responses on visual analog scales (VAS, e.g., Lingjærde and Regine Føreland, 1998; Lesage et al., 2012). We sought to cover essential work factors, an attitude that should represent affect valence (satisfaction), as well as factors that presumably are resistant to context (personality factors). Since we expected that duration of induced affective states may be limited, that a high rate of responding may attenuate the affect produced, and that long periods in the laboratory may be tiring, we limited the number of questions to 41 during each affective state. Therefore, we chose to study single (or two) question items rather than complete scales.

We tested the hypotheses that induced change in affective state alters responding to questions pertaining to: (H1) work tasks (job content), (H2) social interactions at work, (H3) job satisfaction, (H4) pain and health, and (H5) questions from personality trait inventories. In order to elucidate differential effects and to replicate effects, we tested both positive and negative mood induction in all subjects. We expected that inducing positive or negative affective states will alter responding to questions pertaining to work (H1, H2, H3), pain and health (H4), but not to questions pertaining to personality traits (H5).

## Materials and Methods

### Subjects

Employees were recruited from five organizations through written information presented on the organization’s intranet and on the project’s web page. Prior to study participation subjects were informed about the measurements, and that they would undergo challenges consisting of tasks, pictures, music, and text that may influence mood. Screening for criteria of exclusion was done through structured interviews by phone. To be included, participants had to be between the ages of 25 and 50 and work a minimum of 20 h per week. Exclusion criteria were: heart disease, chronic rheumatic diseases, neurological diseases, and mental disorders, as well as prolonged intense pain, addiction to alcohol or drugs, and pregnancy.

Power analysis (G^∗^Power 3, Heinrich Heine Universität, Düsseldorf, Germany) for repeated measures ANOVA with three measure points show that 42 subjects are needed for 80% power to detect 20% change (*p* < 0.05). As previous studies (see Rottenberg et al., 2018) indicate that not all individuals respond to MIPs (i.e., alter their mood), we decided to increase the N by 1/3 to ensure sufficient power to detect change.

Sixty-seven employees took part in the study (women: *N* = 36, men: *N* = 31) from several business sectors (including bank/finance, county administration, health-care, and media). The mean age of the sample was 37.3 years (*SD* = 7.63), range 25–50 years. Mean tenure was 9.0 (*SD* = 8.15) with range 1–30 years and 11.9% reported having leadership responsibilities.

The participants were mailed the NEO-FFI3 questionnaire covering the Five-factor model of personality (McCrae and Costa, 2007) and questionnaires about their work prior to the experimental procedures. These data are beyond the scope of the present article.

The participants were instructed to refrain from alcohol the last 24 h, hard physical activity last 12 h, nicotine and caffeine the last 6 h, and avoid eating the last 3 h before meeting in the laboratory and this was verified by interview prior to entering the laboratory. No women participated in the experiment the last week before their menstrual cycle started and the first 2 days into their cycle. Participants were excluded from the analysis of pain sensitivity due to medication (*n* = 3), caffeine (*n* = 1), nicotine (*n* = 1), day in menstrual cycle (*n* = 1) and one participant was considered an outlier as he was trained in martial arts and in ignoring pain. Furthermore, some participants had complete (*n* = 3) or partial (*n* = 3) missing pain-sensitivity data due to technical malfunction.

### Design

**FIGURE 1:**
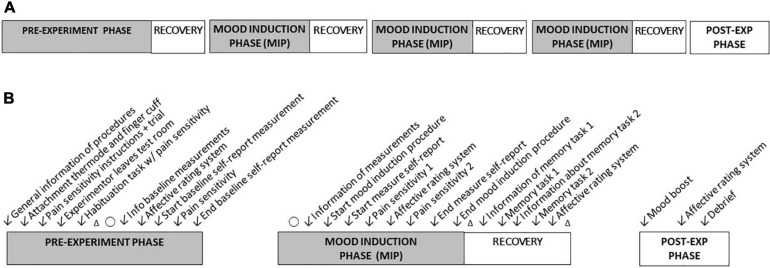
The experimental procedures. **(A)** The general flow of study phases. The order of mood inductions was randomized. **(B)** The order of procedures within study phases. **Δ** = 30 s rest period, O **=** 70 s rest period.

The study had a within-subject cross-over design with five blocks: a pre-experiment phase, three mood induction phases (MIPs), and a post-experiment phase (see Figure 1). All phases were undertaken during a single session in the laboratory (lasting approximately 2 h). The pre-experiment phase included a training session, equipment set up, habituation to the laboratory setting (reading a text aloud for 2 min) and baseline measurements. The manipulation consisted of three blocks corresponding to three mood conditions (i.e., positive, neutral and negative) in random order. After each phase, participants underwent a recovery period with a washout session aimed at resetting participants’ moods.

### Ethical Considerations

The study was approved by the Regional Committees for Medical and Health Research of Norway.

Informed consent was obtained electronically. Participants received a gift card of 1000 NOK (ca € 100) as reimbursement for taking part in the study.

The criteria for exclusion included mental disorders in addition to chronic diseases in order to attenuate the risk of clinically adverse responses (e.g., anxiety, elicit deterioration of depression). We did not ask for medical records. None of the subjects decided to withdraw from the experiment after session start. None of the subjects reported complaints, symptoms, or feeling unwell during or after the experiment.

The post-experiment phase was designed to ensure that participants did not suffer any negative after-effects of the experiment and included a mood boost movie containing a compilation of “feel good” video clips (e.g., funny animal videos) and a debrief interview in which participants were asked about their current mood state and their experiences during the experiment.

### Laboratory Setting

The laboratory was a sound-attenuated room (440 × 286 cm) with one-way mirrors allowing the experimenter, who was seated outside the room, to monitor the participant while participants could not see out. Participants communicated with the experimenter via intercom. During the experiment, participants were seated at a table facing a 34′′ curved LED computer screen (display area 79.8 × 33.6 cm; Samsung S34E790C).

Participants were given thorough pre-recorded instructions before each task. All stimulus material and instructions were presented on a computer screen by software purposely made for the current study and by pre-recorded vocal information. Hence, apart from the initial introduction to the laboratory and the attachment of devises for recording blood pressure and deliver heat stimuli, the experimental procedures were automated to ensure that identical instructions were given to all participants. Hence, experimenter bias was attenuated by minimizing contact between participants and the experimenter. In addition, allowing the participants to been alone in the room may facilitate mood induction as distractions from being observed were attenuated.

### Procedure

#### Mood Induction Procedure

The mood induction procedures involved the presentation of pictures and mood-suggestive music since this combination has been shown to reliably alter mood (e.g., Baumgartner et al., 2006). Sixty-nine pictures (23 for each mood induction) were selected from the International Affective Picture Set (IAPS, Lang, 2005) based on normative data of mood valence and arousal ratings. The criteria for mood valence ratings were set as follows: negative pictures pleasure rating < 3 (mean: 2.43, min: 1.67, max: 3.38), positive pictures pleasure rating > 7 (mean: 7.62, min: 7.04, max: 8.22) and neutral pictures between 4.5 and 5.5 (mean: 5.14, min: 4.7, max: 5.93). As the intent of the experiment was to influence mood valence and not arousal, we sought to minimize effects of concomitant arousal changes. Therefore, only pictures with an arousal rating below six were selected. The music segments are listed in Table 1.

**TABLE 1:** Music segments for mood induction.

**Positive mood condition (5 min 59 s):**
*You’re no different* (by Alan Silvestri from Forrest Gump – Original Motion Picture), *Concerning Hobbits* (by Howard Shore from The Lord of the rings: The Fellowship of the Ring - Original Motion Picture soundtrack), *Meastro* (by Hans Zimmer from The Holiday – Original Motion Picture Soundtrack), *Fairytail* (by Harry Gregson-Williams and John Powell from Shrek – Original Motion Picture Score).

**Negative mood condition (5 min 29 s):**

*All gone* (no escape) (by Gustavo Santaolalla from The Last of Us – Video Game Soundtrack), *Dumbledore’s Farewell* (by Nicholas Hooper from Harry Potter and the Half-Blood Prince – Original Motion Picture Soundtrack), *Adagio for String, op 11* (by Samuel Barber from Greatest Classical Music in Movies), *The North Remembers* (by Ramin Djawadi and The Czech Film Orchestra from Game of Thrones – Music from the HBO series – Season 4).

**Neutral mood condition (6 min 14 s):**

*Call Me* (by Rob Simonsen from Viral – Original Score), *A conspiracy of Good* (by Howard Shore from Denial – Original Motion Picture Soundtrack).

The music was edited with Audacity 2.1.3 (https://audacityteam.org) and the crossfade function was used to create soft transitions and avoid quiet periods between segments (sound files are available on request).

During each mood manipulation, music was played continuously and a subset of nine pictures where presented for 10 s each. Participants were then given three questions from the set of self-report measurements while simultaneously displaying the ninth picture in a smaller format on the right side of the screen. Participants could not continue until all three items were answered. After answering the questions a new full-screen picture was presented for 10 s followed by a new set of questions with the smaller sized picture. This alteration between full-screen pictures and questions continued until all 41 questions were answered. Questionnaire items were presented in a random order for each participant.

#### Washout Procedure

As tasks that draw upon working memory have been found effective in reducing the affective impact of emotion-laden stimuli (Erber and Tesser, 1992; Van Dillen and Koole, 2007; Van Dillen et al., 2009), participants were given two memory tasks in each recovery phase to facilitate mood restoration. The first task participants were presented with a five-digit number that they were asked to retain for 3 s. Two columns of five-digit numbers were then presented on the screen and participants indicated whether the numbers they had retained were in the left column, the right column, or in neither of the columns. A series of 15 such number recollection tasks were given under each recovery phase. In the second task, participants were presented with a black and white picture of 15 objects (e.g., ball, car, skates) that they were asked to memorize. Immediately afterward, they were asked a series of yes-no questions about what they had seen (e.g., “Was there a lamp in the picture?”). The tasks were the same in each recovery phase (see Figure 1), but numbers and objects differed.

#### Pain Sensitivity, Blood Pressure and Heart Rate

Pain sensitivity was tested by a heat-pain threshold test (Somedic Thermal Stimulator, Somedic Sösdala, Sweden). The thermal element (thermode area 25 × 50 mm) was fixed on participants’ volar side of the non-dominant forearm 10 cm from the wrist by an inflatable cuff (15 mmHg). The thermal element default temperature was 30°. Once a thermal stimulation trial was initiated the temperature increased 0.4° per second to a maximum of 48°. The participants were instructed to push the button placed in front of them once “the heat started feeling painful.” They were also informed that the task was not about how much pain they could tolerate. Once participants pushed the button, the thermal stimulation program was stopped and the temperature reverted to default temperature (down speed 10°/second). Participants had no visual clues to indicate the temperature of the thermal element. Pain sensitivity was determined by the time elapsed from onset of the thermal stimulation until the participants pushed the button.

During the habituation and the baseline measurement, the thermal stimulation program was initiated at a random point between 30 and 50 s after the task had started (reading out loud or answering the self-report measures). During the mood induction phases, the first thermal stimulation program was initiated at a random point between 80 and 100 s after the manipulation had started (i.e., when the music started and the first picture was presented). The second thermal stimulation started at a random point between 250 and 270 s. Participants were instructed before the task started that they should push the button when the heat started feeling painful, but they were unaware of the exact time the thermal stimulation program started.

A Finometer model 1 cuff (Finapres Medical Systems, Amsterdam, Netherlands) was placed on the third finger of the subject’s non-dominant hand and calibration were performed prior to the start of the habituation phase of the experiment. The subject rested his/her non-dominant arm on a vacuum cushion on the table throughout the experiment. These data are beyond the scope of the present article and are not reported here.

### Self-Report Measurements

#### Manipulation Check

For assessment of affective changes during MIP, participants were asked to rate their mood valence and arousal, before, during and after MIPs on an affective rating system, the Affect Grid (AG) was adopted. AG is an effective tool when (1) a brief measure is required, (2) detecting frequent short-term changes in mood states, or (3) studies that require a manipulation check (Russell et al., 1989). The Affect Grid has been used for studies of a variety of topics (e.g., Eich et al., 1994; Russell et al., 2016; He et al., 2017; Liapis et al., 2017). Originally, the Affect Grid was a 9 × 9 grid with the axes representing two theoretical dimensions of affect, pleasure, and arousal. For the current study, the Affect Grid was translated into Norwegian and expanded to a 19 × 19 grid to reduce recollection bias. Affect descriptors were placed around the corners according to their relationship with the circumplex model of affect (Posner et al., 2005). During the training session participants were presented with a video with thorough instructions on how to use the affect grid based on the instructions by Russell et al. (1989). Participants indicated their mood by placing a mark somewhere in the grid with their computer mouse.

In order to minimize recollection bias, all other self-report measures were answered on VAS displayed on the computer screen (see Supplementary Figure A1 in Appendix A). The scale was presented as a bar where only the endpoint values were given. Answers were given by clicking with the mouse somewhere on the bar. The position of the response was converted to scale ranging from 1 to 100.

**FIGURE 2:**
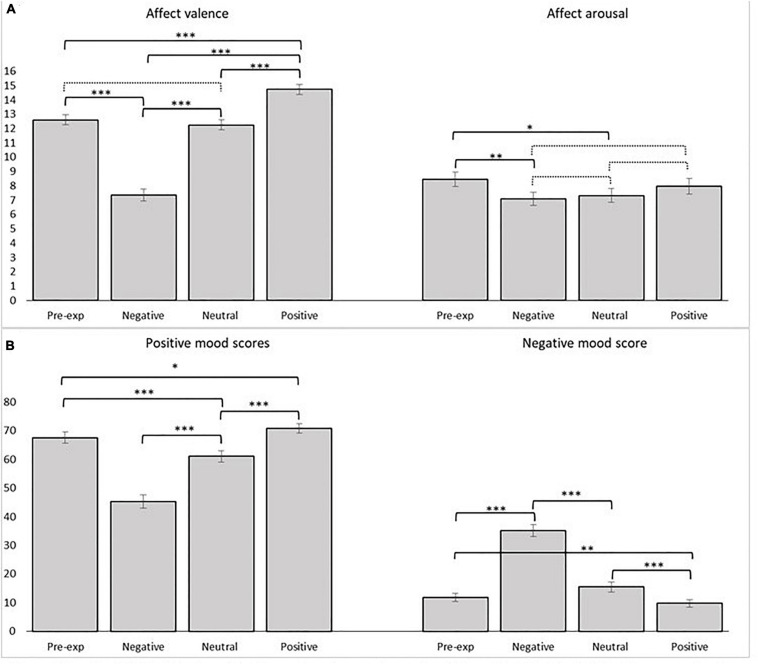
Results of **(A)** the Affective grid rating system valence and arousal and **(B)** emotional state by VAS for the pre-experiment phase, neutral, positive, and negative mood induction phases. Error bars indicate standard error of mean. ^∗^*p* < 0.05; ^∗∗^*p* < 0.01; ^∗∗∗^*p* < 0.001. Dotted lines are non-significant.

#### Affective State

Affective state was measured by the following specific affective states: engaged, sad, relaxed, tired, depressed, tense, uneasy, happy, satisfied, in control, and bored. Each question was phrased “Just now, to what extent are you … (e.g., sad)” and response scales were VAS scales with range (anchoring) “Not at all” and “Maximum.” Indicators of positive and negative mood were created by calculating the mean of happy and satisfied and the mean of sad, depressed, tense, and uneasy, respectively. Both indicators exhibited moderate to adequate internal consistency as assessed by Cronbach’s alphas (range of alphas for the three MIP measurements was 0.64 – 0.81 for the two-item positive- and 0.72 – 0.85 for the four-item negative-mood indicator, respectively).

#### Spontaneous Pain

Pain complaints were the reported intensity of pain in six anatomic regions: arms and/or hands, back, legs, neck and/or shoulders, stomach and head. Each question was phrased “Just now, how much pain do you have in: …. (e.g., Neck and/or shoulders).” The scales ranged from “No pain” to “Insufferable pain.” An overall pain score was created by the mean of scores of the six pain items. Internal consistency was adequate (range of Cronbach’s alphas was 0.77 – 0.81).

#### Perceived General Health

Perceived general health was measured by a single item. “About your health: Generally, would you say that your health is…”. The VAS scale ranged from “Poor” to “Excellent.”

#### Subjective Reports of Work

Work factors were assessed by questions from the General Nordic Questionnaire for Psychological and Social Factors at Work (QPS_Nordic_) with “VAS-response scales Very often or always” to “Very seldom or never.” Each question had the heading “About your job:…”. The QPS_Nordic_ is a thoroughly validated instrument for research and a tool for monitoring and improving working conditions (Dallner, 2000) with Likert-type response scales “very seldom or never,” “rather seldom,” “sometimes,” “rather often,” “very often” for most items.

Two items pertained to quantitative demands: “Is your workload uneven so that your work piles up?”(QD-uneven) and “Do you have too much to do?”(QD-overload). Composite scores were also created by calculating the mean score of the two items. Internal consistency of this composite score was adequate (range of Cronbach’s alphas across the three MIP-conditions was 0.81 –0.87).

Control was measured with single items pertaining to control over work intensity (“Can you yourself determine your work pace”) and decision control (“Can you influence the amount of work that is allocated to you”).

Four items pertaining to social support were included. Two items measured support from leader: “Is your work results appreciated by your immediate supervisor” (LS-appreciation) and “If needed, can you get support and help with your work from your immediate superior?”(LS-help). Support from co-workers was measured with one item: “If needed, can you get support and help with your work from your co-workers?”. In addition, a single item was constructed *de novo* to measure *providing* social support to co-workers: “If they need it, do you give support and help at work to your co-workers?”. A composite score was created by calculating the mean of the two items covering support from leader. The internal consistency of this score was adequate (range of Cronbach’s alphas for the three MIP measurements 0.81 – 0.88).

Role conflict (“Do you have to do things that you think should be done differently”), empowering leadership (“Does your leader encourage you to tell if you have a differing opinion”) and social climate (“How is the work climate in your work unit? Relaxed and comfortable?”) were measured with single items from the QPS_Nordic_.

#### Job Satisfaction

Job satisfaction was measured by two items from the QPS_Nordic_ covering satisfaction with opportunities for personal growth (“How satisfied are you with: That you get to develop yourself personally in your job”; JS-growth) and general job satisfaction (“How satisfied are with: Your job in general – all things considered”; JS-general). The scales ranged from “Very dissatisfied” to “Very satisfied”. A composite score was also created by calculating the mean of the two items. The internal consistency of this composite score was adequate (range of Cronbach’s alphas 0.85 – 0.88).

#### Personality

Five items were selected from the NEO-FFI3 to represent each of the Big Five personality dimensions: neuroticism (“I’m not the type that worries”), extraversion (“I don’t take much pleasure in chatting with people”), agreeableness (“I tend to think the best about people”), openness (“I often try new and foreign food”), and conscientiousness (“I’m a productive person that always gets the job done”). The VAS ranged from “highly disagree” to “highly agree.”

#### Filler Items

In order to attenuate demand characteristics five different filler items were included under each mood phase. Example items are “I like to travel” and “I often listen to music.” These items were also scored with the same VAS scale as all other questions.

### Data Analysis

The statistical analyses were performed with SPSS version 25 (IBM SPSS Statistics, Munich, Germany). Scores were examined for violations of normal distribution prior to analysis. Both the ANOVA and the *t*-test tend to be robust to violations of normality (Gignac, 2019) and most scores were within the suggested cut-off values (Gignac, 2019) for skewness (<2.0) and kurtosis (<9.0). Scores pertaining to self-reported spontaneous pain were, however, severely positively skewed (i.e., the majority of participants’ reported no or very small amounts of pain). Hence, non-parametric tests were used to compare scores between mood phases (i.e., Wilcoxon test) and associations with other constructs (i.e., Spearman’s ρ). Manipulation check was done by repeated measures ANOVA with four levels of mood induction phases: pre-experiment (without induction), neutral, negative, and positive. Non-sphericity was considered by applying Greenhouse–Geisser correction. Effect size estimates were calculated using the online calculator provided by Lenhard and Lenhard (2016). For further examination, planned *post hoc t*-tests were performed.

The effects of mood on self-report measures and pain-sensitivity were analyzed separately by *t*-tests comparing the results in the positive and negative mood phase to the neutral mood phase separately. Since the object of this study was to detect changes in response to manipulations in order to uncover potential sources of bias, we present the *t*-tests (48 tests) with and without correction for family wise error rates (Holm, 1979). There has been critique of conclusions based solely on customary criteria of statistical significance (Wasserstein et al., 2019) and for the present study we based our conclusions on a comprehensive evaluation of the risk of Type I and II errors.

To determine if positive or negative state influenced associations between work factors and outcomes (that is if induced mood influenced common method variance), corresponding correlations (Pearson’s *r*) were computed for the three conditions.

To determine whether including multiple items can attenuate potential reporting biases, both single items and composite scores were analyzed.

## Results

### Manipulation Check – Induction of Affective States

Most of the subjects reported general positive affect valence during the pre-experiment phase, i.e., they started the experiment in a positive mood [mean Affect grid affect valence 12.61 (*SD* = 2.83) and 12.94 (*SD* = 2.74)] during pre-experimental phases (see Figure 2A for a comparison of scores between phases).

Affect valence ratings measured by the Affect grid were significantly different between mood phases [*F*(2.398,158.239) = 93.279, *p* < 0.001; Figure 2A]. The valence rating scores were significantly higher in the positive mood phase (*M* = 14.731, 95% CI = [13.999,15.464]): compared with the pre-experiment phase (*M* = 12.612, 95% CI = [11.922,13.302]), *t*(66) = 5.817, *p* < 0.001, Cohen’s *d* = 0.734, compared with the neutral phase (*M* = 12.254, 95% CI = [11.535,12.972]), *t*(66) = 5.465, *p* < 0.001, Cohen’s *d* = 0.675, and compared with the negative phase (*M* = 7.358, 95% CI = [6.511,8.205]), *t*(66) = 13.124, *p* < 0.001, Cohen’s *d* = 1.498. Also, the valence rating scores in the negative mood phase were significantly lower compared with the pre-experiment phase, *t*(66) = –10.150, *p* < 0.001, Cohen’s *d* = –1.389 and compared with the neutral phase *t*(66) = –11.323, *p* < 0.001, Cohen’s *d* = –1.517. No difference was found between the pre-experiment phase and the neutral phase, *t*(66) = 0.949, *p* < 0.346.

The participants exhibited significantly higher level of arousal during the pre-experiment phase (*M* = 8.463, 95% CI = [7.443,9.482]) compared with the neutral phase (*M* = 7.328, 95% CI = [6.346,8.311], *t*(66) = 2.400, *p* = 0.019) and compared with the negative phase (*M* = 7.104, 95% CI = [6.172,8.037], *t*(66) = 2.767, *p* = 0.007). No differences were found between the positive (*M* = 7.970, 95% CI = [6.873,9.068]), the neutral, or the negative mood phases (all *p*s > 0.05).

The affective state VAS measures supported the findings from the Affect grid rating system (Figure 2B). Positive mood scores differed among mood phases [*F*(2.062,130.069) = 76.998, *p* < 0.001] as did negative mood scores [*F*(2.001,132.082) = 97.786, *p* < 0.001].

Thus, the results of affective rating system and affective state consistently showed that the mood induction procedure used in the present study was effective in inducing corresponding positive, negative, and neutral mood (see Figure 2). Although the intervention had the intended effect on a group level, there may still be individual variability in responsiveness to the mood induction procedures. There are two alternative criteria of non-response to mood induction: (1) those participants not reporting the targeted affect after mood manipulation (state-criterion), and (2) those participants who do not report any *increase* in the target affect after the manipulation (change-criterion) (Rottenberg et al., 2018). We inspected the data for both types of non-responders. The state-criterion of non-response was defined and operationalized as not reporting pleasant feelings on the affective rating system (i.e., scores ≤ 10) during the positive mood induction and not reporting unpleasant feelings (i.e., scores ≥ 10) during the negative mood induction, respectively. The change-criterion of non-response was defined and operationalized as not reporting a change in mood valence on the affective rating system, or change in the unintended direction, compared with the neutral condition. The state-criterion resulted in more non-responders to negative mood induction than the change-criterion, whereas the opposite was found for positive mood induction (see Table 2). This finding is probably due to respondents being in a rather good mood in the pre-experiment phase. For the current study, the change-criterion seems the best indicator of successful manipulation. However, as the two definitions of non-responders may influence results, separate analyses were run with samples based on the two criteria.

**TABLE 2:** Frequency of responders to mood induction.

	**Non-responders to positive MIP**			**Non-responders to negative MIP**			**Responders to both positive and negative MIP**		
	
	***N***	***F***	***M***	***N***	***F***	***M***	***N***	***F***	***M***
State-criteria	5	2	3	9	5	4	53	29	24
Change-criteria	22	10	12	6	4	2	41	24	17

*State-criteria: reported being in the desired mood state. Change-criteria: reported mood valence change in the intended direction. MIP, mood induction procedure; N, total number of participants; F, female; M, male.*

### Effects of Mood Induction on Subjective Reports

Most of the subjective reports of work were unaffected by the mood inductions (Table 3), and there were small effects on variance (see Table 4 for means and SDs). Based on Holm–Bonferroni estimates (Holm, 1979), responding was not significantly affected by transient affective state on any of the questions (lowest *p* = 0.0023 > 0.05/52 for agreeableness).

**TABLE 3:** Results of *t*-tests comparing self-reports in the neutral phase with the mood induction phases (MIP): change criteria.

	**Changes during**		**Changes during**	
	**positive MIP (*n* = 45)**		**negative MIP (*n* = 61)**	
	***t*-value**	***p***	***t*-value**	***p***
Positive mood scores	6.42	<0.001	–8.36	<0.001
Negative mood scores	–4.59	<0.001	10.95	<0.001
**Health**				
General health	–0.98	0.340	–0.74	0.460
**Work factors**				
Job satisfaction – growth	0.35	0.730	2.00	0.050
Job satisfaction – general	–0.69	0.490	–0.28	0.780
Job satisfaction (mean)	–0.24	0.810	1.34	0.180
Quantitative demands – uneven	0.66	0.510	–1.75	0.090
Quantitative demands – overload	–2.34	0.024	–0.93	0.350
Quantitative demands (mean)	–0.99	0.330	–1.61	0.110
Control over work intensity	1.35	0.190	0.40	0.690
Control over decisions	1.74	0.090	1.39	0.170
Control (mean)	1.84	0.070	1.17	0.250
Support from leader – appreciation	–0.62	0.540	0.45	0.650
Support from leader – help	0.60	0.550	0.85	0.400
Support from leader (mean)	0.00	0.990	0.76	0.450
Receiving support from co-workers	–1.36	0.180	–2.13	0.037
Providing support to co-workers	0.42	0.680	–0.23	0.820
Role conflict	–0.50	0.620	0.92	0.360
Empowering leadership	0.42	0.680	1.11	0.270
Social climate	0.23	0.820	0.57	0.570
**Personality**				
Neuroticism	1.74	0.090	–0.45	0.650
Extraversion	–0.69	0.490	1.04	0.300
Agreeableness	3.24	0.002	–1.37	0.180
Openness	–0.09	0.930	–1.33	0.190
Conscientiousness	–0.98	0.340	–0.83	0.410

*Those not reporting changes in affect state (valence) in the intended direction were excluded from the analysis. Positive *t*-values indicate a higher score in the positive/negative mood phase and negative *t*-values indicate a lower score in the positive/negative mood phase.*

**TABLE 4:** Means and standard deviations (all subjects, *N* = 67).

	**Baseline**	**Positive MIP**	**Neutral MIP**	**Negative MIP**
	
	**Mean (Std)**	**Mean (Std)**	**Mean (Std)**	**Mean (Std)**
**Health**				
Spontaneous pain	7.3 (9.0)	5.9 (10.1)	6.7 (9.6)	6.6 (9.3)
General health	80.5 (18.3)	80.4 (19.1)	80.4 (18.8)	80.2 (19.9)
**Work factors**				
Job satisfaction – growth	71.3 (24.1)	71.0 (22.3)	71.2 (21.6)	73.3 (21.3)
Job satisfaction – general	77.0 (21.8)	75.9 (21.4)	76.1 (22.0)	76.0 (21.1)
Job satisfaction (mean)	74.2 (21.2)	73.4 (20.4)	73.7 (20.7)	74.7 (20.0)
Quant. demands – uneven	60.9 (28.8)	58.5 (27.0)	58.9 (27.1)	56.8 (27.7)
Quant. demands – overload	61.9 (26.5)	58.0 (26.3)	60.3 (26.1)	59.1 (27.6)
Quant. demands (mean)	61.4 (24.9)	58.2 (24.4)	59.6 (24.8)	57.9 (25.9)
Control over work intensity	64.3 (25.7)	64.0 (24.9)	61.6 (24.7)	62.1 (24.9)
Control over decisions	55.3 (29.7)	58.3 (26.8)	54.4 (26.6)	56.6 (26.3)
Control (mean)	59.8 (24.0)	61.2 (23.5)	58.0 (24.0)	59.3 (23.5)
Support from leader – appreciation	76.4 (23.1)	75.0 (22.5)	74.6 (22.3)	75.4 (21.4)
Support from leader – help	75.1 (23.5)	75.5 (22.6)	75.0 (22.3)	76.0 (21.7)
Support from leader (mean)	75.7 (20.1)	75.3 (20.7)	74.8 (20.7)	75.7 (20.3)
Receiving support from co-workers	80.6 (20.4)	82.4 (19.6)	82.7 (20.4)	80.9 (19.1)
Providing support to co-workers	90.9 (9.9)	89.8 (10.1)	9.6 (10.1)	89.6 (11.1)
Role conflict	49.7 (26.9)	50.0 (25.9)	49.6 (25.9)	50.5 (25.4)
Empowering leadership	64.9 (27.5)	65.9 (25.8)	64.7 (27.2)	66.2 (25.4)
Social climate	63.4 (24.8)	66.9 (23.8)	66.0 (23.9)	66.9 (23.6)
**Personality**				
Neuroticism	60.9 (34.4)	66.0 (33.1)	63.5 (33.8)	63.0 (33.0)
Extraversion	34.8 (29.9)	32.5 (28.7)	34.7 (28.6)	35.7 (29.3)
Agreeableness	74.8 (19.7)	78.5 (19.0)	76.8 (18.7)	75.8 (20.2)
Openness	74.0 (25.8)	74.9 (25.8)	74.2 (26.0)	73.4 (27.0)
Conscientiousness	81.2 (15.5)	81.0 (14.9)	81.1 (13.7)	80.7 (14.8)

Since the aim of the present study was to detect sources of variance and potential bias, we also tested responses to questionnaire items individually. Keeping in mind the risk of Type I errors, the results demonstrated potential influences on specific factors pertaining to job demands, social support, job satisfaction, and agreeableness. Results of analyses using the change-criterion are presented in Table 3 (results based on the state criterion and on all subjects are presented in the Supplementary Tables B1, C1 in Appendix B, C, respectively).

Common methods variance (CMV) is “systematic error variance shared among variables measured with and introduced as a function of the same method and/or source” (Richardson et al., 2009). If affective state influences CMV, one would expect associations between variables to change with states. Inspection of corresponding correlations between work factor items and outcomes across induced mood conditions revealed that associations were generally similar regardless of affective state (see Supplementary Table D1 in Appendix D). There was no discernible pattern except that the percentage of statistically significant (<0.05) correlations was somewhat higher during positive mood (36.4%) than during neutral (31.8%) and negative mood (31.8%) conditions. The correlation between role conflict and satisfaction with growth was only significant during the neutral condition.

### Changes in Reporting During Positive Mood Induction

Based on the change-criterion participants reported less work overload in the positive mood phase (*M* = 54.47, 95% CI = [46.71, 62.22]) than in the neutral mood phase (*M* = 57.76, 95% CI = [49.95, 65.56]). However, the compound scale of quantitative demands (i.e., mean of the two items) showed no differences between reports in the positive and neutral mood phases. All other items were unaffected by the change toward are more positive mood.

Based on the state-criterion, the report of work overload was in the same direction as with change criterion (i.e., lower in the positive mood phase), albeit no longer significant, *t*(61) = −1.60, *p* = 0.08. This finding suggests that participants had to experience an actual change in mood to affect their reports of work overload.

### Changes in Reporting During Negative Mood Induction

Based on the change-criterion participants reported less support from co-workers in the negative mood phase (*M* = 81.03, 95% CI = [76.27, 85.79]) than in the neutral mood phase (*M* = 83.02, 95% CI = [77.92, 88.11]). All other questions were unaffected by the negative mood change.

Results based on the state-criterion for non-response were similar, except for satisfaction with opportunities for personal growth. The difference in satisfaction with opportunities for personal growth was statistically insignificant for the data based on the change-criterion (*p* = 0.05), but significant based on the state-criterion. This finding is probably a result of the difference in sample size as more participants were excluded based on the change-criterion. The participants reported greater satisfaction during the negative mood induction (*M* = 71.40, 95% CI = [65.81, 76.98]) than in the neutral phase (*M* = 68.88, 95% CI = [63.08, 74.68]).

### Changes in the Reporting of Spontaneous Pain During Mood Induction

Based on the change criterion, Wilcoxon-tests showed non-significant differences between the neutral and the positive phase (*Z* = −1.578, *p* = 0.115) or between the neutral and the negative phase (*Z* = 0.105, *p* = 0.916). Similar results were found based on the state-criterion (neutral vs. positive: *Z* = −1.689, *p* = 0.091, neutral vs. negative: *Z* = −0.128, *p* = 0.898) and when including all subjects (neutral vs. positive: *Z* = −1.421, *p* = 0.155, neutral vs. negative: *Z* = −0.472, *p* = 0.637). These results indicate that the mood inductions did not influence self-reported measures of spontaneous pain.

### Heat-Pain Sensitivity

Pain sensitivity was lower (i.e., it took longer for the participants to push the button) in the second or third test within each mood phase (see Supplementary Figure E1 in Appendix E for more details). Lower pain sensitivity in the following tests may be due to a peripheral desensitization or habituation to pain within each mood phase. Therefore, the mean score of the two measurements within each mood phase was calculated to compare pain sensitivity between mood phases. Pain sensitivity did not differ between the positive and neutral mood phase [change-criterion: *t*(38) = 0.70, *p* = 0.49; state-criterion: *t*(48) = 1.05, *p* = 0.30] nor between the negative and neutral mood phases [change-criterion: *t*(36) = 1.58, *p* = 0.12; state-criterion: *t*(48) = 1.48, *p* = 0.15]. Thus, the findings regarding pain-sensitivity align with the self-reports of spontaneous pain in that the mood inductions did not influence pain perception.

## Discussion

The aims of the current study were to determine whether affective state affects the reporting of work factors, subjective health, and questions for assessment of personality traits. The experimental manipulations successfully induced positive and negative mood with little change of arousal in the majority of the participants. In general, subjective reports were little or not affected by transient affective states even if taking non-response to the manipulation into account. This finding was replicated and pertains to both positive and negative mood states. However, we cannot rule out that this conclusion only applies to questions that are phrased for neutral valence. If one consider each question item separately, i.e., disregard the high risk of Type I error due to the high number of statistical tests, it seems that affect may influence subjective reports pertaining to (i) negative valence (job demands “too much to do”), (ii) attitudes (job satisfaction), and (iii) social interactions (social support from co-workers and agreeableness).

To our knowledge, this is the first study of the effects of experimentally induced mood on self-report measurements of working conditions so comparable studies are lacking. The present results are in accordance with the finding that subjective reports of questionnaires pertaining to marital distress were insensitive to depressed mood induced by music and autobiographical recall (Heene et al., 2007). The appraisal of one’s work should be familiar or typical and the past judgment may be directly accessible. Hence, on a theoretical level, the finding is in accordance with the AIM of affect and judgment (Forgas, 1995), which posits that affect infusion should not influence judgments “whenever the target is either familiar or typical, a relevant past judgment can be directly accessed in memory, and there is little internal or external demand for reprocessing” (“direct access processing”).

Of the two items covering quantitative demands, only work overload (“too much to do”) was influenced by mood change (however not statistically significant by Holm–Bonferrini correction for family wise error rate), showing a lower level during the positive emotion condition, but little change during the negative condition. Work overload was the only item included in the current study for which participants implicitly perform an evaluation of their capacity to handle the requirements of their job, i.e., their coping with the challenges at work. It seems reasonable to hypothesize that measurements of the appraisal of coping with challenges are prone to bias by affective state. However, the response to the work-overload question was not significantly influenced by the negative affective state. The two job-demands questions belong to a quantitative job-demands scale (Dallner, 2000). Comprehensive scales may attenuate effects of transient affective state on individual items if the measured construct *per se* is not influenced.

The report of satisfaction with opportunities for personal growth (“satisfied with getting to develop my own personality through my job”) increased during the negative affective state. Hence, data obtained by questions pertaining to valenced factors seems susceptible to affective state and vulnerable to bias. There is no simple explanation why negative mood can result in a more positive appraisal of one’s job and slightly lower demands. There were no apparent effects of the manipulations on the level of arousal and one can only speculate that negative mood may influence reporting of valenced questions by (i) attention mechanisms due to the discrepancy between the present affective state and the rating of an implicitly positive factor or (ii) as a compensatory mechanism to counteract the negative mood (“mood repair”). However, it seems that neutral wording of questions and response alternatives is of pivotal importance for ensuring low sensitivity to affective states.

Responses to the question of availability of social support from co-workers was influenced negatively by negative mood (although not statistically significant by Holm–Bonferrini correction for family wise error rate). Strikingly, the item representing the agreeableness-dimension of the NEO-FFI3 was vulnerable to transient affective state with an increase in responses during positive mood. It seems plausible that the type of mood-induction procedure may be part of the explanation since several of the pictures shown portrayed people. On the other hand, transient affective states did not alter the reporting of the extraversion-question. There was no effect on the reporting of availability of support from one’s immediate superior. Possibly, employees perceive this an aspect of leadership rather than social interactions. Previous studies have found that induced depressed mood by reading self-referent statements (Velten, 1968) was associated with the reporting of lower social support (Cohen et al., 1988) and high levels of agreeableness and extraversion are associated with perceived social support (for review see Swickert and Owens, 2010). Emotion regulation may be intertwined with social interactions (Lopes et al., 2005). Taken together these findings suggest that questions addressing social interactions with other persons may be vulnerable to bias induced by affective state.

None of the health-outcome measures were affected by mood. Previous studies have provided ambiguous results regarding the effect of mood on self-reported health and pain. Croyle and Uretsky (1987) reported two studies showing that experimentally induced negative mood caused people to perceive their health more poorly and to increase their reports of recent physical symptoms. However, subsequent studies have failed to reproduce these findings (Barger et al., 2007). Abele and Hermer (1993) found that self-appraisal of health was more negative under induced negative mood, but not more positive under positive mood induction. Subjective general health may represent central self-conceptions and may thereby be insensitive to mood influence, i.e., there is differential sensitivity to mood (Sedikides, 1995). Constantinou et al. (2013) induced transient mood by pictures and found an interactive effect of unpleasantness and high arousal on symptom reporting, but only in high habitual symptom reporters. Reports of spontaneous pain (sum of six regions) were slightly lower during the positive mood state, but this effect was statistically insignificant.

Based on our non-response analyses, the current study also suggests that some individuals may be more sensitive to changes in mood than others. There are a variety of factors that may be the source of these sensitivities, such as neuroticism and extroversion (Blackburn et al., 1990; Blagrove and Akehurst, 2001), self-esteem (Greenberg et al., 1992), sleep deprivation (Gruber and Cassoff, 2014), and affective state prior to the experiment (e.g., level of anxiety or depression; Blackburn et al., 1990; Scherrer and Dobson, 2009). Several of these factors have been associated with subjective reports of working conditions. For instance, neuroticism and extroversion have been associated with reporting more negative and positive working conditions (Judge et al., 2002), respectively. More research is needed to determine whether affective state is a mediator in the relationship between individual factors and reporting behavior.

### Methodological Considerations

The present study has several methodological strengths. The study had a strict procedure for instructions to abstain from activities that may influence psychological and physiological responding (physical activity, nicotine, caffeinated beverages, alcohol) and a thorough interview to verify this prior to laboratory testing. Menstrual cycle phase was controlled. All subjects were employees with full-time jobs, hence work environment questions were relevant for them. We excluded individuals with health conditions that may influence response to mood induction and work ability.

Both experimental sequences of MIPs and order of questions within each MIP were randomized. The experimental manipulations were presented automatically by a computer program and the experimenter observed the subjects from an adjoining room. Pre-experiment procedures ensured that the participants habituated to the test environment prior to mood manipulations. Furthermore, our results indicate that the washout procedure was effective in restoring participants’ mood between mood phases (for more details see Supplementary Figure F1 in Appendix F). This finding lends support to previous studies (Erber and Tesser, 1992; Van Dillen and Koole, 2007; Van Dillen et al., 2009) showing that loading working memory prevents mood-congruent processing and promotes distractions from the current mood. Hence, it seems reasonable to conclude that the experimental design did not introduce sources of method bias.

The pictures shown were selected among IAPS-pictures (Lang, 2005) with low arousal rating < 6 and we found no significant differences between the three conditions in self-reported arousal. The pre-experiment arousal report was higher than subsequent reports showing that habituation to the test environment was adequate. Habituation to the testing context is important for obtaining valid results.

The standard response scales of all questions tested by the present experiment were five-point scales (“very seldom or never,” “rather seldom,” “sometimes,” “rather often,” “very often” for most items of the QPS_Nordic_, and “highly disagree” to “highly agree” for items of the NEO-FFI3). This type of scale may allow recall of recent previous responses. In order to (i) attenuate recall of previous responses, (ii) increase sensitivity to detect differences, and (iii) to produce interval-level measurements (Reips and Funke, 2008) the subjects responded by placing a mark (with the computer mouse) at 262 mm horizontal visual analog scales (VAS-scales) with verbal anchor descriptors at each extreme end. Question responses based on VAS may not equal responses based on Likert-type scales, but for the present objectives of detecting changes in response to manipulation this is not relevant. The measurements of affective states with VAS-scales showed changes which corresponded to those of the affect grid, suggesting that the VAS-method was adequate for this study.

Any study of effects of manipulations may be subject to demand characteristics (Orne, 1962), commonly described as the cues and context and expectations of the situation which influences the participants to generate assumptions of what is investigated and expectations of how to behave and to anticipate findings. This mechanism may be a concern since the observed effects on induced mood state could have been inflated due to participants’ cooperative attitudes and demand-characteristics, i.e., the observed effects on mood consist of reporting mood changes to adhere to what subjects assume are the experimental demands. However, studies of this non-specific effect have primarily addressed mood induction by subjects reading self-reference mood statements, usually referenced as the Velten procedure (Velten, 1968; Buchwald et al., 1981; Kwiatkowski and Parkinson, 1994). A study that sought to determine the role of demand characteristics did find that mood shifts were not artifactual (Polivy and Doyle, 1980).

Although we cannot rule out demand-characteristics effects, we believe that this effect was of limited importance. First, although for ethical reasons participants were informed that they would be subjected to a task that could influence their mood before taking part in the study, they were blind to the order of the manipulations, and the exact same instructions were given prior to each manipulation (i.e., positive, neutral, and negative) with no human interaction. Meta-analytical examination of the effect of other mood induction procedures suggests that participants report changes in mood both in conditions where they are aware and unaware (i.e., studied using cover stories) of the mood manipulation (Larsen and Sinnett, 1991). Second, participants could not get any visual clues from the experimenter during the experiment as the mood manipulation procedure was automated and participants were seated alone in the test room. Furthermore, some of the participants were classified as non-responders to the mood manipulations suggesting that the participants, in general, did not feel pressure to report mood changes. Finally, participants were not informed about the objective of the study (i.e., whether mood may influence their self-report measures) prior to the experiment. Even if mood reports were somewhat inflated due to demand-characteristics, this mechanism cannot explain the lack of effects of transient affective state on most work factors and the rather specific effects on reporting social interactions and valenced factors.

The questions tested by the present experiment were items selected from validated scales of work factors (Dallner, 2000) and personality traits (McCrae and Costa, 2007). It may be argued that complete scales have known psychometric properties and may be more robust to transient states. Since the object of the present study was to detect change and since effects were small or negligible, we maintain that testing items rather than complete scales does not influence the conclusions drawn.

#### Ecological and External Validity

The manipulation stimuli, pictures, and music, are not related to the subjects’ work tasks or their work environments. Furthermore, the experiments were undertaken in a laboratory and procedures of attaching devices for heat stimulation and blood pressure measurements probably were a novel experience for the subjects. Therefore, the transient emotions produced did not pertain to or relate to specific situations at their work. Since the objective of the present study was to determine the effects of transient affective states on subjective reports, not the effects of cognitive factors, these considerations should not invalidate the present conclusions.

The current study aimed to induce moderate transient mood changes to resemble everyday mood intensities that a wide variety of individuals experience. Kwiatkowski and Parkinson (1994) found that induced and natural depressive moods differed in the recall of target words and we cannot rule out that more severe mood changes (e.g., being in a clinically depressive state) may bias reporting. Hirschfeld et al. (1983) found that being in a clinically depressed state strongly influenced some but not all personality measures, Reich et al. (1986) concluded that state anxiety and depression are potential confounding factors in personality measurements, while Lewis et al. (1995) found that experimentally induced mood affected optimism and pessimism scores in women.

In order to cover several work factors, the current study only investigated one or two questions of each scale or construct. Therefore, one cannot determine whether the findings generalize to the measurement of the complete scales or constructs (e.g., agreeableness). Moreover, all work questions had frequency-response scales (“seldom of never” – “often or always”) and one should probably refrain from generalizing conclusions to questionnaire formats with response scales indicating agreement (“fully agree” – “fully disagree” or “true” – “untrue”).

## Conclusion and Implications

The present study shows that transient affective states at the time of responding generally produce small and insignificant effects on subjective reporting of common psychological work factors when measured with questions with neutral valence. Transient affective states primarily may influence responding to questions pertaining to valence (“satisfied with …”, “too much to do”) and social interactions (social support from co-workers, agreeableness), but effects were small (<4% difference between means and <13% in standard deviations). Therefore, questions for measuring psychological and social work factors are rather resistant to moderate affective states if items are phrased in a neutral way that does not imply valence. Naturally, adequate procedures for assessing validity and reliability must be followed (e.g., Price, 2017). It seems reasonable to conclude that in general, responses to neutral-valence worded questions are little influenced by transient positive or negative affective states, with the possible exception of questions addressing social interactions (social support and agreeableness).

Introducing measures of the current affective state into questionnaire instruments seems a simple method for detecting the risk of mood-induced bias. However, using a state measure as a correction factor in analyses introduces the risk of eliminating a mediating factor. Since the present study indicates that questions with neutral valence are quite resistant to transient affective states at the time of responding to questionnaires, careful examination of affective content of questions is crucial for obtaining reliable measures (see also Chen and Spector, 1991).

Specific attitudes, cognitive schema, or basic beliefs in addition to abilities may influence how work is perceived and appraised. Mental health may influence the reporting of job demands (Dalgard et al., 2009). Hypothetically, assumptions of consequences of one’s work exposures may influence perceptions and appraisal. While the present study points to small or negligible effects of transient affective states, there is a need for studies of cognitive factors inherent in measurement contexts (e.g., information, expectations, beliefs, attitudes) which may influence and bias responding to questionnaires and interviews.

## Data Availability Statement

The raw data supporting the conclusions of this article will be made available by the authors, without undue reservation.

## Ethics Statement

The studies involving human participants were reviewed and approved by The Norwegian regional committees for medical and health research – the Regional Ethics Committee, REK sør-øst. The patients/participants provided their written informed consent to participate in this study.

## Author Contributions

SK formulated the research questions and developed the methods. KA developed the methods (including developing the software for standardized communication with research subjects), participated in the data collection (screening of subjects) and analyzed the data. KA and SK wrote the manuscript. Both authors contributed to the article and approved the submitted version.

## Conflict of Interest

The authors declare that the research was conducted in the absence of any commercial or financial relationships that could be construed as a potential conflict of interest.
