# Supplementary figures and images for: The Influence of Affective State on Subjective-Report Measurements: Evidence From Experimental Manipulations of Mood
Source: Front Psychol. 2021 Feb 18;12:601083. doi: 10.3389/fpsyg.2021.601083 (PMC7930079; doi:10.3389/fpsyg.2021.601083)

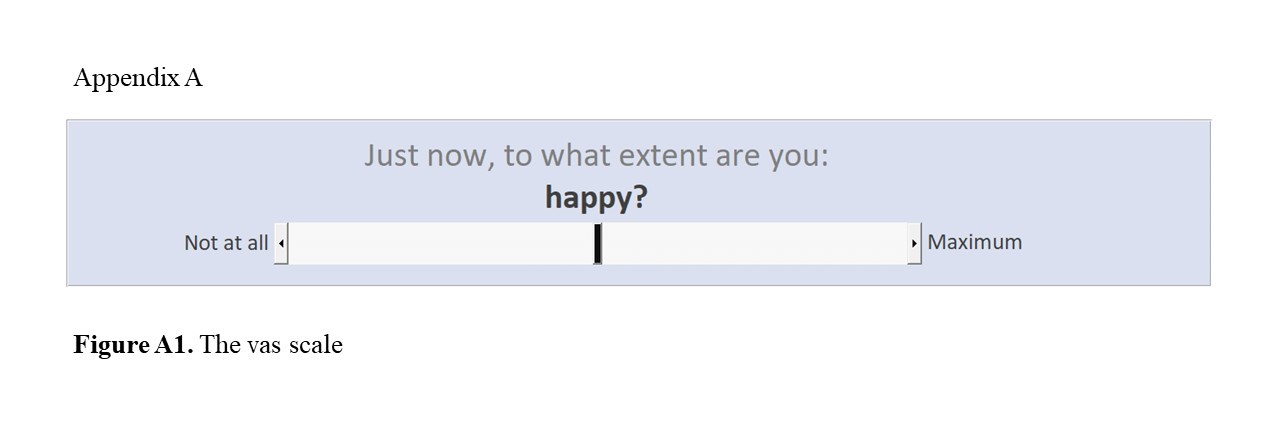

Supplement: Supplementary file 1 [file Image_1.JPEG]

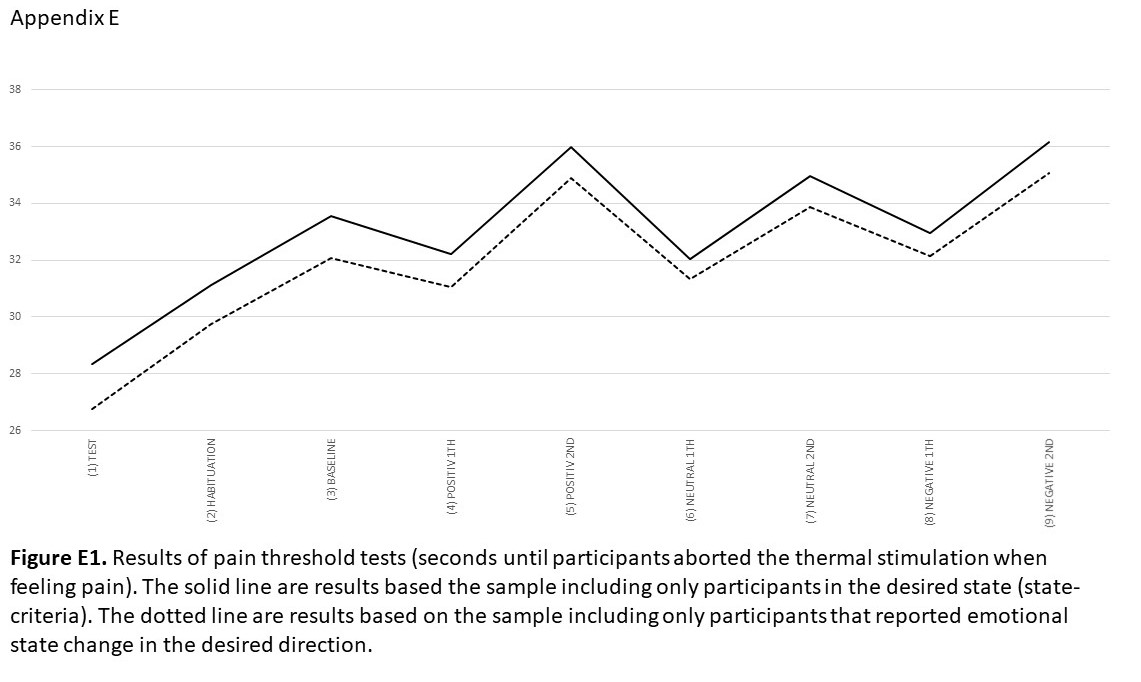

Supplement: Supplementary file 2 [file Image_2.JPEG]

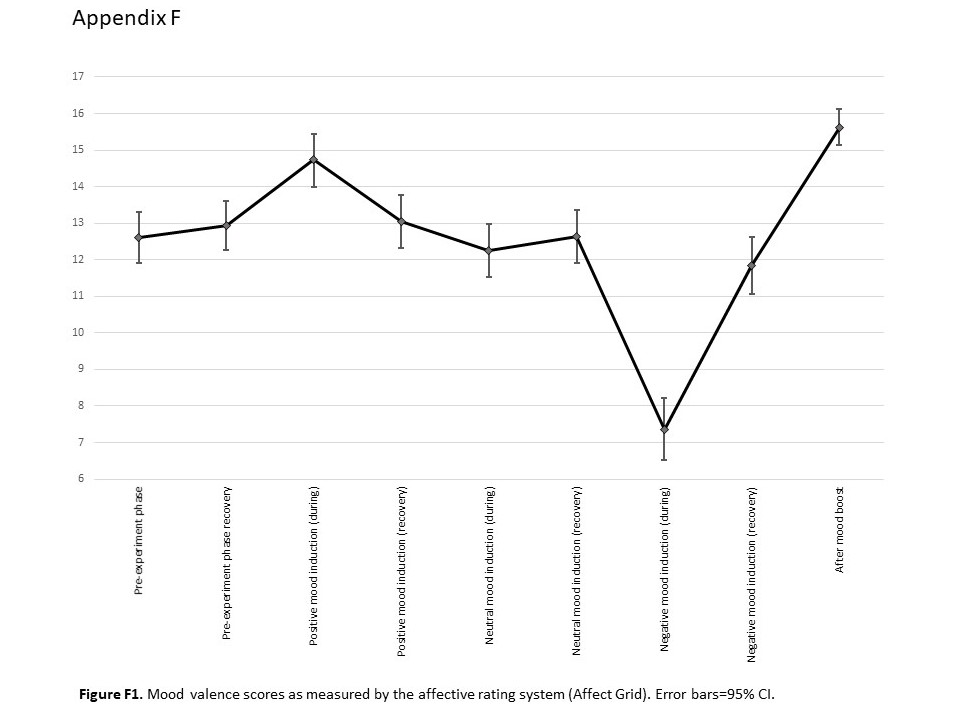

Supplement: Supplementary file 3 [file Image_3.JPEG]
