# Supplementary material for: The Influence of Affective State on Subjective-Report Measurements: Evidence From Experimental Manipulations of Mood
Source: Front Psychol. 2021 Feb 18;12:601083. doi: 10.3389/fpsyg.2021.601083 (PMC7930079; doi:10.3389/fpsyg.2021.601083)
Supplement: Supplementary file 4 [file Table_1.docx]

Appendix B

| Table B1  *Results of t-tests comparing self-reports in the neutral phase with the mood induction phases (MIP): state criteria* | | | | | | |
| --- | --- | --- | --- | --- | --- | --- |
|  |  | Changes during  positive MIP  (n=62) | |  | Changes during  negative MIP  (n=58) | |
|  |  | t-value | p |  | t-value | p |
| *Health parameter* |  |  |  |  |  |  |
| General health |  | -0.21 | .83 |  | -0.60 | .55 |
| *Work factors* |  |  |  |  |  |  |
| Job satisfaction - growth |  | -0.32 | .75 |  | 2.19 | .04 |
| Job satisfaction - general |  | -0.48 | .64 |  | -0.11 | .91 |
| Job satisfaction (mean) |  | -0.55 | .58 |  | 1.58 | .12 |
| Quantitative demands - uneven |  | -0.06 | .96 |  | -1.53 | .13 |
| Quantitative demands - overload |  | -1.60 | .12 |  | -0.85 | .40 |
| Quantitative demands (mean) |  | -1.20 | .24 |  | -1.41 | .16 |
| Control over work intensity |  | 1.15 | .25 |  | 0.20 | .84 |
| Control over decisions |  | 1.78 | .08 |  | 1.51 | .14 |
| Control (mean) |  | 1.81 | .08 |  | 1.11 | .27 |
| Support from leader - appreciation |  | 0.45 | .65 |  | 0.37 | .71 |
| Support from leader - help |  | 1.40 | .16 |  | 0.83 | .41 |
| Support from leader (mean) |  | 1.08 | .28 |  | 0.69 | .50 |
| Receiving support from co-workers |  | -0.36 | .72 |  | -2.10 | .04 |
| Providing support to co-workers |  | 1.20 | .24 |  | -0.39 | .70 |
| Role conflict |  | -0.14 | .90 |  | 0.90 | .37 |
| Empowering leadership |  | 0.82 | .42 |  | 1.18 | .24 |
| Social climate |  | 0.93 | .35 |  | 0.68 | .50 |
| *Personality* |  |  |  |  |  |  |
| Neuroticism |  | 1.62 | .11 |  | 0.40 | .69 |
| Extraversion |  | -1.19 | .24 |  | 1.02 | .31 |
| Agreeableness |  | 2.84 | <.01 |  | -1.68 | .10 |
| Openness |  | 1.22 | .23 |  | -1.40 | .17 |
| Conscientiousness |  | -0.18 | .86 |  | -1.00 | .32 |
| *Note.* Those not in the desired affect state were excluded from the analyses. Positive t-values indicate a higher score in the positive/negative mood phase and negative t-values indicate a lower score in the positive/negative mood phase. | | | | | | |
