# Supplementary material for: The Influence of Affective State on Subjective-Report Measurements: Evidence From Experimental Manipulations of Mood
Source: Front Psychol. 2021 Feb 18;12:601083. doi: 10.3389/fpsyg.2021.601083 (PMC7930079; doi:10.3389/fpsyg.2021.601083)
Supplement: Supplementary file 5 [file Table_2.docx]

Appendix C

| Table C1  *Results of t-tests comparing self-reports in the neutral phase and mood induction phases (MIP): all subjects* | | | | | | |
| --- | --- | --- | --- | --- | --- | --- |
|  |  | Changes during  positive MIP  (n=67) | |  | Changes during  negative MIP  (n=67) | |
|  |  | t-value | p |  | t-value | p |
| *Health parameter* |  |  |  |  |  |  |
| General health |  | -1.76 | .08 |  | -0.27 | .79 |
| *Work factors* |  |  |  |  |  |  |
| Job satisfaction - growth |  | -0.17 | .86 |  | 1.85 | .07 |
| Job satisfaction - general |  | -0.28 | .78 |  | -0.18 | .86 |
| Job satisfaction (mean) |  | -0.31 | .76 |  | 1.37 | .18 |
| Quantitative demands – uneven |  | -0.34 | .74 |  | -1.54 | .13 |
| Quantitative demands - overload |  | -1.74 | .09 |  | -0.91 | .36 |
| Quantitative demands (mean) |  | -1.53 | .13 |  | -1.46 | .15 |
| Control over work intensity |  | 1.20 | .23 |  | 0.26 | .80 |
| Control over decisions |  | 2.28 | .03 |  | 1.42 | .16 |
| Control (mean) |  | 2.17 | .03 |  | 1.08 | .29 |
| Support from leader - appreciation |  | 0.33 | .74 |  | 0.55 | .58 |
| Support from leader - help |  | 0.46 | .65 |  | 1.21 | .23 |
| Support from leader (mean) |  | 0.49 | .62 |  | 1.01 | .32 |
| Receiving support from co-workers |  | -0.33 | .75 |  | -2.16 | .04 |
| Providing support to co-workers |  | 0.37 | .71 |  | 0.05 | .96 |
| Role conflict |  | 0.23 | .82 |  | 0.64 | .53 |
| Empowering leadership |  | 1.00 | .32 |  | 1.54 | .13 |
| Social climate |  | 0.87 | .39 |  | 1.07 | .29 |
| *Personality* |  |  |  |  |  |  |
| Neuroticism |  | 1.66 | .10 |  | -0.39 | .70 |
| Extraversion |  | -1.01 | .32 |  | 0.74 | .46 |
| Agreeableness |  | 2.43 | .02 |  | -1.03 | .31 |
| Openness |  | 1.22 | .23 |  | -1.13 | .27 |
| Conscientiousness |  | -0.16 | .87 |  | -0.72 | .47 |
| *Note*. Including all participants. Positive t-values indicate a higher score in the positive/negative mood phase and negative t-values indicate a lower score in the positive/negative mood phase. | | | | | | |
