# Supplementary material for: The Influence of Affective State on Subjective-Report Measurements: Evidence From Experimental Manipulations of Mood
Source: Front Psychol. 2021 Feb 18;12:601083. doi: 10.3389/fpsyg.2021.601083 (PMC7930079; doi:10.3389/fpsyg.2021.601083)
Supplement: Supplementary file 6 [file Table_3.docx]

Appendix D

| Table D1  *Correlations between work-factor questions that are commonly treated as independent and dependent factors, respectively, as a function of mood induction phases (all subjects are included, N = 67).* | | | | |
| --- | --- | --- | --- | --- |
| Work factors | Outcome factors | Positive mood | Neutral mood | Negative  mood |
| Quantitative demands – uneven | General health | -.240 | -.167 | -.157 |
|  | Spontaneous pain | .196 | .211 | .241* |
|  | Job satisfaction - growth | .114 | .108 | .168 |
|  | Job satisfaction - general | .000 | .043 | .066 |
| Quantitative demands - overload | General health | -.191 | -.215 | -.214* |
|  | Spontaneous pain | .058 | .157 | .098 |
|  | Job satisfaction - growth | .140 | .056 | .057 |
|  | Job satisfaction - general | .137 | .129 | .113 |
| Role conflict | General health | -.145 | -.079 | -.118 |
|  | Spontaneous pain | .098 | .016 | .091 |
|  | Job satisfaction - growth | -.200 | -.374** | -.231 |
|  | Job satisfaction - general | -.313** | -.358** | -.323** |
| Control over work intensity | General health | .250* | .082 | .116 |
|  | Spontaneous pain | -.009 | .076 | -.078 |
|  | Job satisfaction - growth | .246* | .123 | .217 |
|  | Job satisfaction - general | .143 | .075 | .084 |
| Control over decisions | General health | .066 | .142 | -.017 |
|  | Spontaneous pain | -.070 | -.084 | -.029 |
|  | Job satisfaction - growth | .198 | .207 | .124 |
|  | Job satisfaction - general | .037 | .086 | -.034 |
| Support from leader - help | General health | .201 | .201 | .144 |
|  | Spontaneous pain | -.318** | -.280* | -.215 |
|  | Job satisfaction - growth | .473** | .396** | .236 |
|  | Job satisfaction - general | .476** | .393** | .275* |
| Support from leader - appreciation | General health | .205 | .144 | .140 |
|  | Spontaneous pain | -.266* | -.227 | -.255* |
|  | Job satisfaction - growth | .516** | .450** | .368** |
|  | Job satisfaction - general | .510** | .473** | .428** |
| Getting co-worker support | General health | .001 | .110 | .149 |
|  | Spontaneous pain | -.155 | -.175 | -.147 |
|  | Job satisfaction - growth | .146 | .244* | .146 |
|  | Job satisfaction - general | .273* | .292* | .244* |
| Giving co-worker support | General health | .176 | .134 | .141 |
|  | Spontaneous pain | -.116 | -.017 | -.170 |
|  | Job satisfaction - growth | .063 | .054 | .076 |
|  | Job satisfaction - general | .121 | .055 | .131 |
| Empowering leadership | General health | .284* | .272* | .221 |
|  | Spontaneous pain | -.319** | -.199 | -.262* |
|  | Job satisfaction - growth | .347** | .372** | .293* |
|  | Job satisfaction - general | .363** | .296* | .312* |
| Social climate | General health | .192 | .187 | .251* |
|  | Spontaneous pain | -.158 | -.093 | -.173 |
|  | Job satisfaction –growth | .453** | .295* | .324** |
|  | Job satisfaction - general | .515** | .417** | .444** |
| *Note.* Spearman’s rho was used to estimate correlations between spontaneous pain and work factor items as the pain measure violated the assumptions of normal distribution. Other correlations was tested estimated with Pearson’s r. * denotes two-tailed test statistically significant at p < .05, ** denotes two-tailed test statistically significant at p < .01. | | | | |
